# Tobacco and the Parotid Gland

**Published:** 1870-12

**Authors:** 


*Tobacco and the Parotid Gland.*—The "Herald of Health" in an editorial on the perverted use of saliva says, "tobacco greatly excites the parotid gland so that when this substance is in the mouth it pours out a great amount of saliva to dilute and wash away the offending substance. In this case it becomes lost. It may be an interesting fact to some of our readers to know that so great is the demand for saliva in the mouths of tobacco chewers, that nature has to provide for this increase by enlarging the parotid gland. Surgeons when dissecting this gland in tobacco chewers, have often noticed this to be the case. If the tobacco did any good, there would be no harm in this; but as it does not, the harm comes from wasting our forces unnecessarily. Persons who have more force than they know what to do with may not be much harmed by this waste; but this number is few. Most of us have none too much strength, even when all of it is saved for best uses, so the folly of habits such as that of using tobacco wastes our strength, and is so filthy that it ought to condemn itself if it produced no physiological injury."

